# Genome-wide identification and functional analysis of the *TIFY* gene family in response to drought in cotton

**DOI:** 10.1007/s00438-016-1248-2

**Published:** 2016-09-17

**Authors:** Ge Zhao, Yun Song, Caixiang Wang, Hamama Islam Butt, Qianhua Wang, Chaojun Zhang, Zuoren Yang, Zhao Liu, Eryong Chen, Xueyan Zhang, Fuguang Li

**Affiliations:** State Key Laboratory of Cotton Biology, Institute of Cotton Research, Chinese Academy of Agricultural Sciences, Anyang, 455000 China

**Keywords:** *TIFY* gene family, *Gossypium* spp., Phylogenetic tree, Expression analysis, Drought assay

## Abstract

Jasmonates control many aspects of plant biological processes. They are important for regulating plant responses to various biotic and abiotic stresses, including drought, which is one of the most serious threats to sustainable agricultural production. However, little is known regarding how jasmonate ZIM-domain (JAZ) proteins mediate jasmonic acid signals to improve stress tolerance in cotton. This represents the first comprehensive comparative study of TIFY transcription factors in both diploid A, D and tetraploid AD cotton species. In this study, we identified 21 *TIFY* family members in the genome of *Gossypium arboretum*, 28 members from *Gossypium raimondii* and 50 *TIFY* genes in *Gossypium hirsutum*. The phylogenetic analyses indicated the *TIFY* gene family could be divided into the following four subfamilies: TIFY, PPD, ZML, and JAZ subfamilies. The cotton TIFY genes have expanded through tandem duplications and segmental duplications compared with other plant species. Gene expression profile revealed temporal and tissue specificities for *TIFY* genes under simulated drought conditions in *Gossypium arboretum*. The *JAZ* subfamily members were the most highly expressed genes, suggesting that they have a vital role in responses to drought stress. Over-expression of *GaJAZ5* gene decreased water loss, stomatal openings, and the accumulation of H_2_O_2_ in *Arabidopsis thaliana*. Additionally, the results of drought tolerance assays suggested that this subfamily might be involved in increasing drought tolerance. Our study provides new data regarding the genome-wide analysis of *TIFY* gene families and their important roles in drought tolerance in cotton species. These data may form the basis of future studies regarding the relationship between drought and jasmonic acid.

**Electronic supplementary material:**

The online version of this article (doi:10.1007/s00438-016-1248-2) contains supplementary material, which is available to authorized users.

## Introduction

Cotton is the most important fiber and oil crop worldwide, and its production is threatened by abiotic stresses, such as drought and salinity stresses (Grayson 2013). The International Service for the Acquisition of Agri-biotech Application reported that in Australia, the area on which cotton was grown decreased by 200,000 hectares between 2013 and 2014 because of an ongoing drought (http://www.isaaa.org/). Therefore, breeding for drought-resistant cotton should be a high priority for plant biotechnology programs. Phytohormones, such as auxin, gibberellin, and abscisic acid, considerably affect plant growth, development, aging, dormancy, and stress resistance. Jasmonic acid (JA) is considered as a new plant hormone, and there is interest regarding whether it can be used to increase stress tolerance in cotton (Kumar et al. 2014).

Jasmonic acid is a small oxylipin signaling molecule that is ubiquitous in the plant kingdom. It regulates plant responses to various biotic and abiotic stresses, such as wounding, UV light, water deficit, pathogens, and ozone. It is also involved in a wide range of plant developmental processes, including pollen and stamen development, vegetative growth control, anthocyanin accumulation, and senescence (Conconi et al. 1996; McConn and Browse 1996; Creelman and Mullet 1997; Rao et al. 2000; Berger 2002; Schommer et al. 2008; Cheng et al. 2009; Ren et al. 2009; Qi et al. 2011; Wang et al. 2013).

COI1 is the F-box protein subunit of SCF^COI1^ (ubiquitin ligase complex), which is a member of the Skip/Cullin/F-box family of E3 ubiquitin ligases that functions as a central conserved component of the JA signaling pathway in *Arabidopsis thaliana* and tomato (Xie et al. 1998; Chini et al. 2007; Thines et al. 2007). The substrate specificity of SCF^COI1^ is mainly based on the specificity of the F-box protein. The substrate is ubiquitinated by E3 ubiquitin ligases, and subsequently degraded by the 26S proteasome (Moon et al. 2004).

The jasmonate ZIM-domain (JAZ) proteins were identified as a substrate of SCF^COI1^ by Thines et al. (2007). The JAZ proteins are localized to the nucleus, although they do not contain a DNA-binding domain (Grunewald et al. 2009). These proteins repress jasmonate responses by interacting with the basic helix-loop-helix (bHLH) transcription factors MYC2 and MYC3, which bind to DNA sequences and regulate downstream gene expression (Lorenzo et al. 2004; Chini et al. 2007; Grunewald et al. 2009; Cheng et al. 2011). SCF^COI1^/JAZ/MYC is a three-component core complex of the JA signaling pathway. Because SCF^COI1^ is highly conserved, JA is believed to have diverse functions that depend on JAZ proteins and MYC transcription factors.

In 2007, proteins containing a zinc finger protein expressed in inflorescence meristem (ZIM) domain were discovered in a study of JA responses in *Arabidopsis thaliana* (Nishii et al. 2000). Because of the presence of a conserved motif (i.e., TIFYXG), the ZIM domain was renamed as the TIFY domain (Vanholme et al. 2007). The JAZ proteins belong to the TIFY family, and have been identified as a key regulator of JA (Chini et al. 2007; Thines et al. 2007). The well-characterized *TIFY* genes have been studied for their vital role in JA-related mechanisms. Several studies demonstrated that when plants are treated with JA and other stresses, such as pathogens, drought, cold, or salinity, the *TIFY* genes provide protection by inducing a differential negative feedback loop mechanism that returns plants to their normal state (Chini et al. 2007; Thines et al. 2007; Chung et al. 2008; Demianski et al. 2012).

Previous studies on JAZ proteins in the model plant *A. thaliana* confirmed their importance as regulators of JA responses. Recent studies have focused on rice and grapevines. Several rice and grape genes that might influence tolerance to various types of abiotic stress, such as drought, salinity, and low temperature, have been suggested (Ye et al. 2009; Zhang et al. 2012). Currently, there is limited information regarding JA expression and the functions of *TIFY* family genes in cotton.

Cotton is valued for its role in fiber production, and is a vital commodity in the global economy. The availability of a sequenced *Gossypium* spp. genome enables genome-wide identification and computational analysis of the *TIFY* gene family. In this study, we report the analysis of the *TIFY* genes from *Gossypium arboretum*, *Gossypium raimondii* and *Gossypium hirsutum* pertaining to genomic organization, gene structures and the phylogenetic tree analysis. Although *G. arboreum* (A genome) produces inferior fiber, it exhibits drought tolerance, disease resistance, and can grow in desiccated areas (Mehetre et al. 2003). So, the TIFY gene family expression in *G. arboretum* is observed under drought stress conditions and conduct drought tolerance assays. In addition, the results indicate the over-expression of *GaJAZ5* genes enhance drought tolerance in cotton by affecting various stress-related pathways. To the best of our knowledge, this is the first report of a genome-wide analysis of *TIFY* genes in cotton. The potential roles of *TIFY* genes in drought stress signaling and JA signaling pathways should be further investigated.

## Materials and methods

### Identification and motif analysis of the *TIFY* genes

The DNA and corresponding amino acid sequences of 21 *TIFY* genes were retrieved from the Genome A Database (http://cgp.genomics.org.cn/), based on the published mRNA-seq data (Zhang et al. 2013). A local BLASTP search was completed to identify complete *TIFY* genes, using *A. thaliana* TIFY sequences as queries. A BLASTP search (e value: 1e^−10^) was used to obtain a dataset of TIFY proteins, including 28 *Gossypium raimondii*, 50 *Gossypium hirsutum*, 16 *Theobroma cacao* sequences, 18 *A. thaliana* sequences, 14 *Ricinus communis* sequences, 25 *Populus trichocarpa* sequences, and 34 *Glycine max* sequences downloaded from the GenBank (http://ncbi.nlm.nih.gov/genbank) and Phytozome (http://phytozome.jgj.doe.gov/pz/portal.html) databases. To identify all members of the *TIFY* gene family, previous *TIFY* gene sequences were analyzed using the Simple Modular Architecture Research Tool (SMART; http://smart.embl-heidelberg.de/) (Letunic et al. 2006) and Pfam (http://pfam.janelia.org/) (Bateman et al. 2004).

### Sequence alignment and phylogenetic analysis

Multiple amino acid sequence alignments of TIFY proteins from *G. arboreum*, *G.raimondii*, *G. hirsutum, T. cacao*, *A. thaliana*, *R. communis*, *P. trichocarpa*, and *G. max* were completed with the ClustalW program (Larkin et al. 2007). Additionally, phylogenetic trees were constructed using MEGA 5.05 software, and evolutionary distances were determined using the p-distance method and 1000 bootstrap samples (Wei et al. 2014), which established the reliability of each tree. Amino acid identities were confirmed using JEMBOSS 1.5. Synonymous (d_S_) and non-synonymous (d_N_) substitution rates were calculated with the PAL2 NAL web server (http://www.bork.embl.de/pal2nal/#RunP2N), which uses the CODEMAL program of PAML (Suyama et al. 2006).

### Structure analysis and chromosomal localization of the *TIFY* genes

We used SMART (http://smart.embl-heidelberg.de/smart/set_mode.cgi?NORMAL=1) to identify *TIFY* domains (Letunic et al. 2012), and the MEME (http://meme.nbcr.net/meme/) program was used to predict motifs. The exon–intron structures of the *TIFY* genes were determined by comparing the coding sequences and corresponding genomic sequences. The Gene Structure Display Server (http://gsds.cbi.pku.edu.cn) (Hu et al. 2015) was used to analyze the exon–intron structures. Data from the Genome Database and Mapchart software were used to predict the chromosomal locations of the *TIFY* genes. Adobe Photoshop software was used to visualize the distribution of *TIFY* genes on the chromosomes.

### *Gossypium arboreum TIFY* gene expression analysis

To determine *TIFY* gene expression levels and assess their association with drought resistance in leaves, roots, and stems, *G. arboreum* cv. ‘Shixiya’ seedlings were treated with polyethylene glycol (PEG) to simulate drought conditions and then analyzed by quantitative reverse transcription polymerase chain reaction. After the emergence of three to four leaves, plants were treated with 17 % PEG 6000 or water (control) for 6 h. Roots, stems, and leaves were collected every 30 min for subsequent qRT-PCR analyses. The sample was obtained from at least three individual plants for each stage. An RNAprep Pure Plant kit (Tiangen, China, Beijing) was used to extract total RNA. Reverse transcription reactions were completed using 0.5 μg RNA and an iScript cDNA synthesis kit (Bio-Rad, USA). The ABI Power SYBR Green PCR Master Mix (Applied Biosystems, USA) was used with 5 ng template. First-strand cDNA was synthesized from DNaseI-treated total RNA using 1 μl iScript reverse transcriptase (TaKaRa, Japan), 0.5 μg RNA, and 4 μl 5× Reaction Mix. Samples were incubated at 25 °C for 5 min, followed by 42 °C for 30 min, 85 °C for 5 min, and finally storage at 4 °C. The qRT-PCR was conducted in an optical 384-well plate using an ABI PRISM 7500 real-time PCR system (Applied Biosystems). Gene-specific primers were designed for *TIFY* gene family members (Table S1). The SYBR Green qRT-PCR reaction consisted of 5 μl 2× SYBR Green PCR buffer, 0.5 μl primers, and 5 ng templates. The final volume was adjusted to 10 μl with double-distilled H_2_O. The PCR program was as follows: 50 °C for 2 min; 95 °C for 10 min; 40 cycles at 95 °C for 15 s and 60 °C for 1 min. Data were processed using the 2^−ΔΔCt^ method (Livak and Schmittgen 2001). The experiment was repeated three times. A heat map was prepared using Cluster 3.0.

### Cloning of the *Gossypium arboreum TIFY* genes

Total RNA was extracted from *G. arboreum* seedling tissues using Trizol (Sigma-Aldrich) according to the manufacturer’s instructions. Samples were reverse transcribed using an oligo (dT) primer and SuperScript II reverse transcriptase (Promega, USA). The resulting product was used as a PCR template to amplify the predicted *TIFY* open reading frames using PrimeSTAR DNA Polymerase. The *TIFY* cDNA regions were cloned into a T-simple vector (TaKaRa). The accuracy of all clones was confirmed by sequencing. *TIFY*-specific primers were designed, synthesized, and used to clone the genes. For plant transformations, the *TIFY* cDNA samples were inserted into the modified pSuper1300 plant transformation vector (provided by Zhen Su from China Agricultural University) under the control of the cauliflower mosaic virus 35S promoter.

### *Arabidopsis thaliana* transformation and screening of transgenic plants

The *TIFY*-pSuper1300 constructs were introduced into *Agrobacterium tumefaciens* strain GV3101 cells, which were then used to transform *A. thaliana* plants according to the floral dip method (Clough and Bent 1998). The resulting T_3_ generation was used for all subsequent experiments. Total DNA was extracted from *G. arboreum* tissues with the DNAprep Pure Plant kit (Tiangen, China). Primers were synthesized (forward primer: TTGAATAGATACGCTGACACGC; reverse primer: CTATCCCTTTCTCTTCTCGA) and used along with the following PCR program: 94 °C for 5 min; 32 cycles at 94 °C for 40 s, 60 °C for 40 s, and 72 °C for 50 s.

### Effect of polyethylene glycol on *TIFY* gene expression

The effects of PEG on *TIFY* expression were evaluated by qRT-PCR. Samples were collected from *A. thaliana* plants treated with PEG for 4 h. Total RNA was extracted from the samples and reverse transcribed as described to generate cDNA for qRT-PCR analyses.

### Drought tolerance assay

Drought tolerance was assessed by transferring 7-day-old plants cultured in Petri dishes to pots (10-cm diameter) filled with a vermiculite:perlite mixture [1:1.5 (v/v)]. Seedlings were cultured for 2 weeks with constant watering before drought conditions were simulated. After 17 days without water, all plants were simultaneously rewatered, and plant re-growth was scored 2 days later. Six plates for individual transgenic lines were used in each repeated experiment, with every plate consisting of 35 g vermiculite:perlite mixture.

### Water loss assay

Leaves were harvested from 9-week-old mutant and wild-type (WT) seedlings at the rosette stage. Changes in fresh weight (FW) over time were monitored using an electronic balance, and the rate of water loss was calculated as the loss in fresh weight. Ten plants of each transgenic and WT line were analyzed in this assay, which was replicated three times (Duan et al. 2012). Fully expanded leaves were cut from the plants, and the FW was recorded immediately. Then, the leaves were immersed in distilled water for 0.5 h and the turgid weight was recorded. Finally, the dry weight (DW) was recorded after drying for 6 h at 23 °C in an oven. The water loss rate (%) was calculated as follows: (FW − DW) × 100/FW.

### Ratio of open to closed stomata

Leaf samples (without veins or edges) were harvested from 7-day-old mutant and WT seedlings, and treated with 10 % PEG 6000 for 3 h. Samples were observed under a light microscope and the open and closed stomata were counted.

### Hydrogen peroxide analysis

Leaves from 10-day-old mutant and WT seedlings were treated with 10 % PEG 6000 for 12 h. The H_2_O_2_ content was then determined. Leaves and stems were harvested from 4-week-old mutant and WT seedlings at the rosette stage following treatment with 10 % PEG 6000 or water (control) for 12 h. Samples were treated with the REAL EnVision Detection System (Peroxidase/DAB+, Rabbit/Mouse) (Dako) solution for 8 h at 28 °C, destained with ethanol, and then observed under a stereoscopic microscope.

## Results

### Genome-wide identification of *TIFY* genes

Twenty-one *TIFY* family genes were obtained from the cotton Genome A Database (http://cgp.genomics.org.cn). All of the genes had different Garb numbers resulting from mRNA-seq data (Zhang et al. 2013). Then, we used a Blast program (ftp://ftp.ncbi.nlm.nih.gov/blast/executables/release) based on the multiple sequence alignment results of Arabidopsis TIFY protein sequences to identify TIFY genes in *Gossypium arboretum*. We used databases containing *Gossypium raimondii* and *Gossypium hirsutum* genome sequences of the cotton research institute of Chinese academy of agricultural sciences, anyang, Henan (Li et al. 2015). After removing redundant sequences, we identified 28 *TIFY* genes in the *G. raimondii* and 50 *TIFY* genes in the *G. hirsutum* with an e value of less than one. We named the genes according to their chromosomal locations and genetic structures (Table 1, Table S2, Table S3 and Fig. S1).

**Table 1:** The *TIFY* family genes in *Gossypium arboretum*

Name	GeneIDa	Chr	CDs	No. of amino acid	Group A ID	Group D ID	Group AD ID	Identity (%)
GaJAZ1	KT312820	1	687	228	Cotton_A_05658	Cotton_D_10015133	CotAD_18763	98.98
GaJAZ2	KT312821	1	690	229	Cotton_A_14056	Cotton_D_10037287	CotAD_40859	97.05
GaJAZ3	KT312822	2	1092	363	Cotton_A_36376	Cotton_D_10019921	CotAD_67052	99.02
GaJAZ4	KT312824	3	759	252	Cotton_A_11862	Cotton_D_10010230	CotAD_22999	92.18
GaJAZ5	KT312825	4	813	270	Cotton_A_18896	Cotton_D_10023394	CotAD_62298	99.18
GaJAZ6	KT312829	6	3345	1114	Cotton_A_01448	Cotton_D_10030605	CotAD_21832	31.93
GaJAZ7	KT312830	7	732	243	Cotton_A_00049	Cotton_D_10017986	CotAD_24822	93.58
GaJAZ8	KT312831	8	594	197	Cotton_A_10012	Cotton_D_10001798	CotAD_02206	93.75
GaJAZ9	KT312832	8	786	261	Cotton_A_09418	Cotton_D_10039035	CotAD_46116	93.68
GaJAZ10	KT312835	9	723	240	Cotton_A_27840	Cotton_D_10037314	CotAD_00351	99.17
GaJAZ11	KT312836	9	363	120	Cotton_A_02904	Cotton_D_10031542	CotAD_75527	98.81
GaJAZ12	KT312837	10	1095	364	Cotton_A_36075	Cotton_D_10005893	CotAD_41943	98.91
GaJAZ13	KT312838	10	360	119	Cotton_A_12336	Cotton_D_10033602	CotAD_27478	98.89
GaZML1	KT312827	6	867	288	Cotton_A_30531	Cotton_D_10039389	CotAD_32387	96.92
GaZML2	KT312828	6	945	314	Cotton_A_15821	Cotton_D_10038217	CotAD_35015	96.08
GaZML3	KT312833	8	891	296	Cotton_A_24823	Cotton_D_10011723	CotAD_68174	98.28
GaZML4	KT312834	8	1062	353	Cotton_A_24824	Cotton_D_10011724	CotAD_67271	99.72
GaZML5	KT312840	13	939	312	Cotton_A_17214	Cotton_D_10029545	CotAD_65649	95.35
GaPPD1	KT312823	3	1080	359	Cotton_A_16991	Cotton_D_10020204	CotAD_67677	91.94
GaPPD2	KT312826	5	1248	415	Cotton_A_41299	Cotton_D_10031607	CotAD_75535	93.01
GaTIFY	KT312839	10	1284	427	Cotton_A_11516	Cotton_D_10016076	CotAD_50802	95.70

**Fig. 1:**
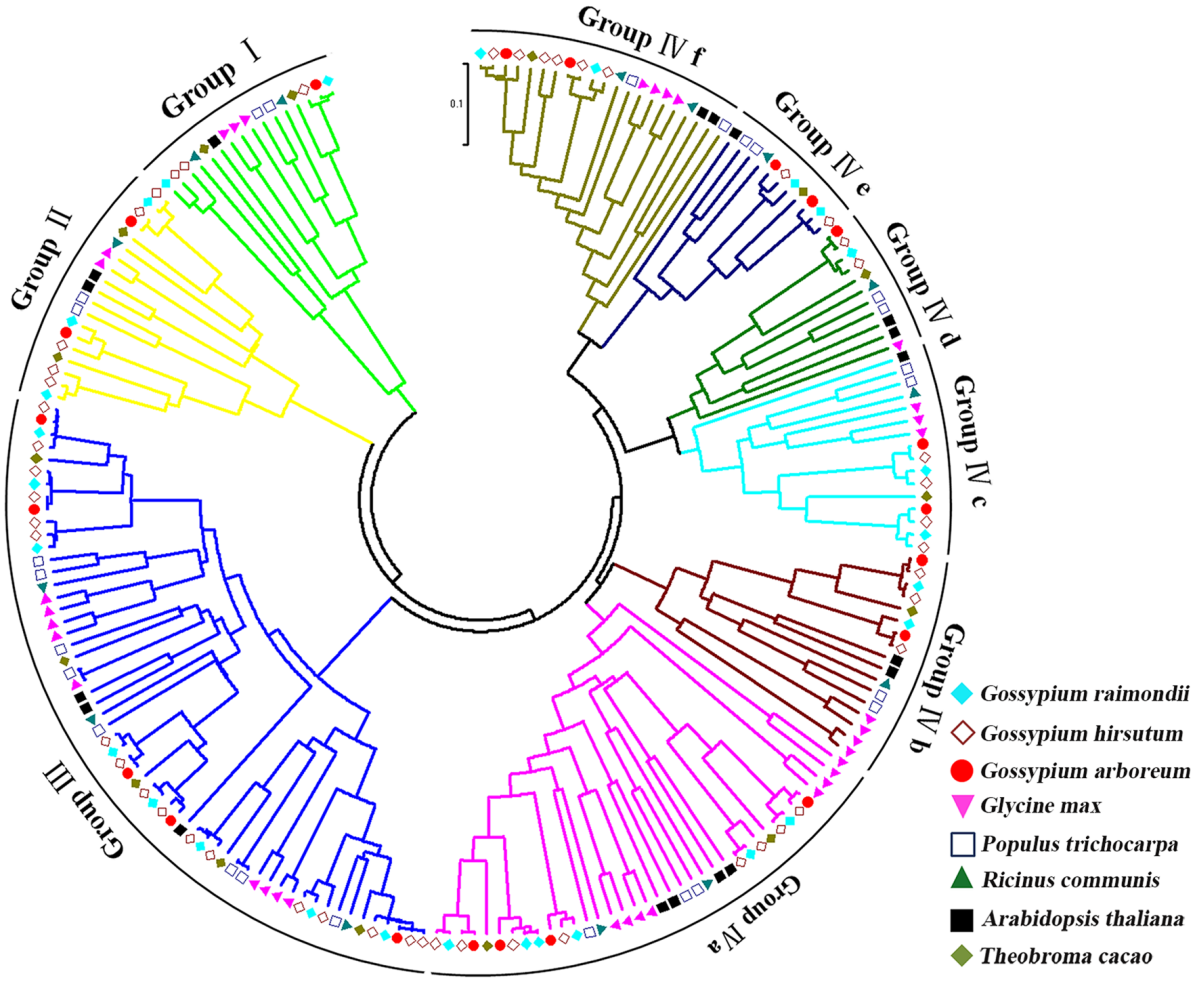
Unrooted neighbor-joining phylogenetic tree of *TIFY* genes from *Gossypium arboreum*, *Gossypium raimondii*, *Gossypium hirsutum*, *Arabidopsis thaliana*, *Theobroma cacao*, *Glycine max*, *Ricinus communis*, and *Populus trichocarpa. Group I* ZML subfamily, *Group II* TIFY subfamily, *Group III* PPD subfamily, *Group IV* JAZ subfamily

The 21 *TIFY* family genes had a high similarity index (i.e., 91.94–99.72 %) among *G. arboreum* (genome A), *G. raimondii* (genome D), and *G. hirsutum* (genome AD) genomes (Table 1; Fig. 1). This similarity indicated the genes were highly conserved during the evolution of *Gossypium* spp., except for unigene 3984 (Fig. S2). These findings indicated that the *TIFY* genes likely diverged evolutionarily from early terrestrial plants, which involved considerable mutations that resulted in functional divergence. Because of the similarity among the A, D, and AD genomes, and to avoid the complexity of *G. hirsutum* (tetraploid cotton), we used *G. arboreum* to study the relationships among *TIFY* family genes, JA, and drought conditions.

### Phylogenetic analysis and classification of the *TIFY* gene family

**Fig. 2:**
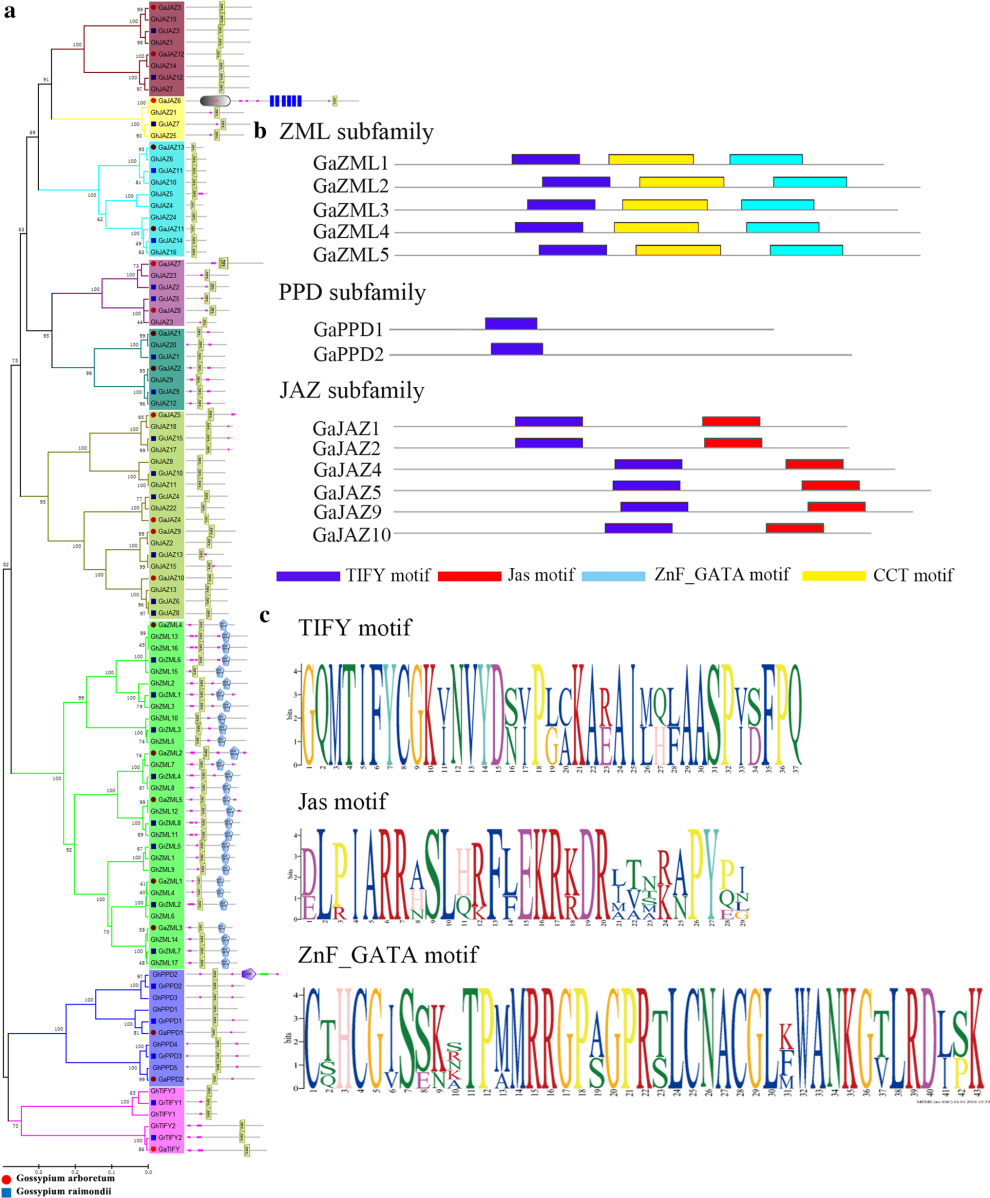
Phylogenetic relationships and gene structures of *Gossypium* spp. *TIFY* genes. **a** Four subfamilies (Group I–IV) are indicated with *different colors*. Group IV is divided into six subgroups with *different colors*. A *schematic diagram* of amino acid motifs is provided on the *right side*. **b** Distribution of the conserved motifs in the ZML, PPD, and JAZ subfamilies identified by MEME. Motif locations are indicated at the *bottom*. **c** TIFY, Jas, and ZnF_GATA motifs

To assess the phylogenetic relationships of the *TIFY* family among *G. arboreum*, *G. raimondii*, and *G. hirsutum*, *A. thaliana*, *T. cacao*, *G. max*, *R. communis*, and *P. trichocarpa*, 206 full-length amino acid sequences were used to construct a phylogenetic tree (Fig. 1). The results indicated that all of the analyzed species diverged from a common ancestor, with cotton diverging the most. The amino acid sequences could be divided into four groups. Using the cotton protein sequences as an example, the protein sequences containing the TIFY and Jas motifs were predicted to belong to the JAZ subfamily. The proteins lacking a conserved PY structure at the C-terminus were predicted to belong to the PPD subfamily. The proteins with TIFY and CCT motifs, and a ZnF_GATA motif, were predicted to belong to the ZML subfamily. The protein contained just the TIFY motif, which suggested it belonged to the TIFY subfamily (Fig. 2a). These classification results were consistent with those based on domain compositions, as described above (Fig. 2b). The structure of the main motif is presented in Fig. 2c. We identified 28 *TIFY* genes in the *G. raimondii* genome, which were classified into the *TIFY* (two genes), *PPD* (three genes), *ZML* (eight genes), and *JAZ* (15 genes) groups (Table S2) (He et al. 2015). Additionally, 50 *TIFY* genes were identified in the *G. hirsutum* genome, and were classified into the *TIFY* (three genes), *PPD* (five genes), *ZML* (17 genes), and *JAZ* (25 genes) groups (Table S3). The *G. arboreum TIFY* genes were divided into four subfamilies: *TIFY* (one gene), *PPD* (two genes), *ZML* (five genes), and *JAZ* (13 genes). According to the phylogenetic tree, the JAZ subfamily could be divided into six subgroups and were associated with *A. thaliana*, *T. cacao*, *G. max*, *R. communis*, and *P. trichocarpa* (Table 2).

**Table 2:** List of the number of *TIFY* family genes

	Group I	Group II	Group III	Group IV					
				IVa	IVb	IVc	IVd	IVe	IVf
*Gossypium arboretum*	5	1	2	4	2	2	1	2	2
*Gossypium raimondii*	8	2	3	6	2	2	1	2	2
*Gossypium hirsutum*	17	3	5	8	3	4	2	2	6
*Arabidopsis thaliana*	3	1	2	4	2	1	2	1	2
*Theobroma cacao*	5	1	2	3	1	1	1	1	1
*Glycine max*	9	3	2	8	4	3	1	0	4
*Ricinus communis*	3	1	1	3	1	1	1	1	2
*Populus trichocarpa*	8	2	2	3	2	2	2	2	2

### Analysis of the exon–intron structure of the *TIFY* genes

**Fig. 3:**
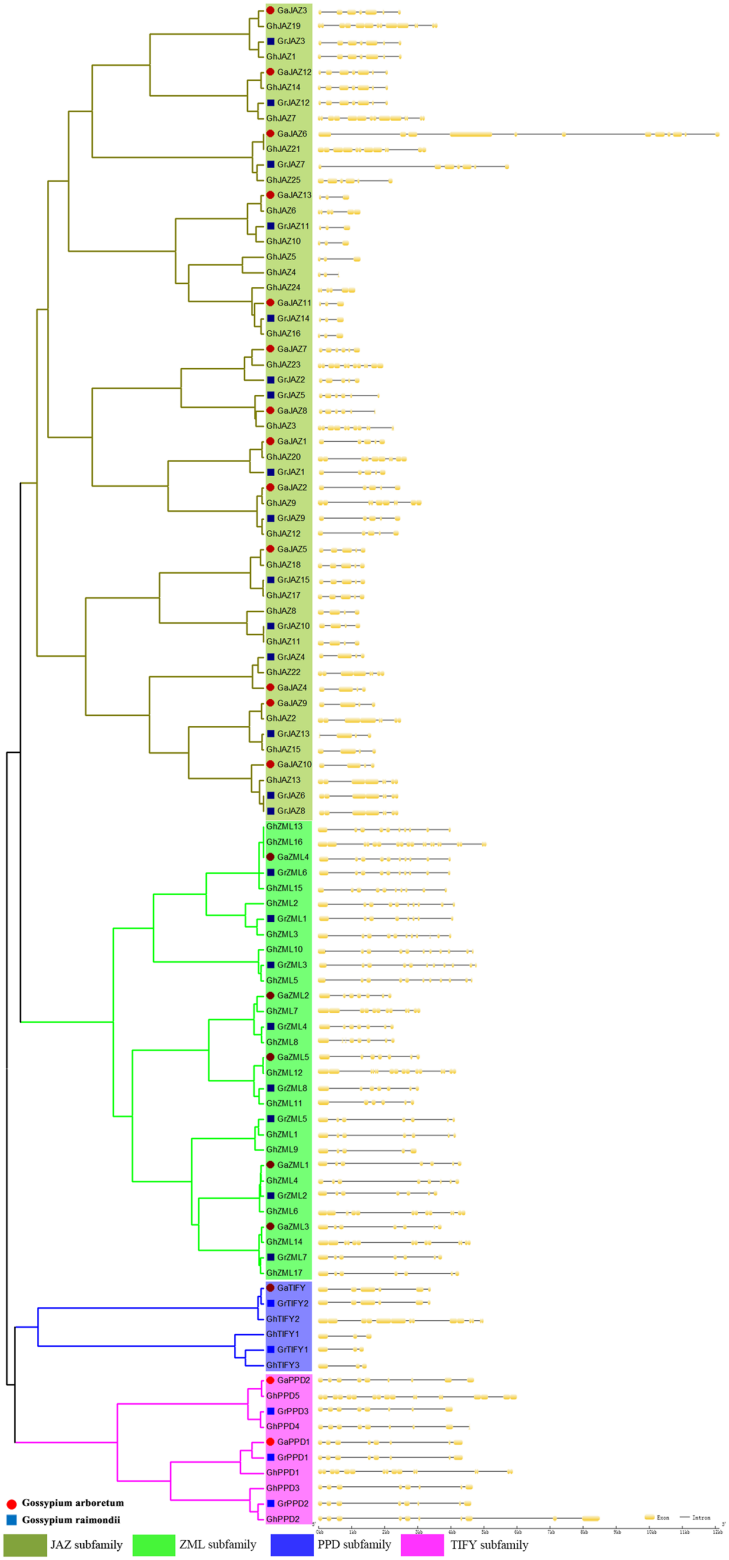
Exon–intron structures of the *Gossypium* spp. *TIFY* gene family

To determine the origin of TIFY paralogs, we analyzed the structures of the *TIFY* genes. Compared with the phylogenetic results, the results of the analyses of exon–intron structures suggested that Groups I–IV had undergone exon deletions. The exon–intron analysis shows that gap of exons in *G. arboretum* and *G. raimondii* was smaller, but most the number of exons in tetraploid cotton (*G. hirsutum*) was the sum of exons in *G. arboretum* and *G. raimondii* or similar to *G. arboretum* and *G. raimondii*. Take exon–intron analysis in *G. arboretum* as examples, we made a concrete analysis of exon–intron. Analyses of the exon–intron structure revealed there were 3–12 exons in each of the analyzed *TIFY* genes in *G. arboreum*. Eight *TIFY* genes had six or seven exons, and six *TIFY* genes had four or five exons. Additionally, *GaJAZ6* had the most exons and introns, possibly because of gene duplication events (Fig. 3). Furthermore, 80 % of the ZML subfamily members had seven exons, suggesting that the genomes were highly conserved during evolution. However, the number of exons among JAZ subfamily members ranged from 3 to 12, suggesting that considerable changes had taken place during evolution. Therefore, the proteins in the JAZ subfamily likely have different functions because of a frame shift. This conservation of exon and intron numbers correlated well with the clades identified in the phylogenetic tree for each group. This strongly supports the close evolutionary relationships of *TIFY* genes in Groups I–IV.

### Chromosomal location and evolutionary history of the *TIFY* gene family

Twenty-one *TIFY* genes were mapped to 11 *G. arboreum* chromosomes. There were thirty-eight *TIFY* genes mapped to 13 chromosomes and the other 12 genes distribution on the 9 scaffolds in *G. hirsutum*, which were not assemble into chromosomes. All the *TIFY* genes *G. raimond*ii were located on the scaffolds, which was different with the published articles (He et al. 2015). Take diploid cotton *G. arboretum* as examples, each chromosome contained at most four genes (Fig. S1). It is believed that the duplication mechanisms (e.g., tandem, segmental, and whole-genome duplications) in eukaryotes are very similar. These mechanisms caused gene families to expand, as observed in *G. arboreum* (Wang et al. 2012). To infer the possible relationships between *TIFY* genes and potential gene duplications, we analyzed the occurrence of tandem and large-scale segmental duplications during the evolution of this gene family (Table S4). Among the 21 *TIFY* genes, six paralogs were identified according to the chromosomal locations. By analyzing the similarities and sequences of the six paralogs, we determined that only one gene had undergone a tandem duplication event (i.e., *GaJAZ2/1*), implying that this type of duplication was not prevalent during the evolution of the *TIFY* gene family. Five segmental duplication events (i.e., *GaJAZ3/12*, *GaJAZ9/10*, *GaZML3/1*, *GaZML4/2*, and *GaJAZ11/13*) were detected in *G. arboreum* (Fig. S1). This result suggested that the expansion of the *G. arboreum TIFY* gene family was mainly due to segmental duplication events.

Nucleotide substitutions in protein-coding genes can be classified as synonymous or non-synonymous. To assess the evolutionary history of the *TIFY* gene family, *d*_S_ and *d*_N_ values were calculated using PAL2NAL. These values can be used to determine whether protein-coding genes have undergone a selection process (de Las et al. 2004). We estimated the *d*_N_/*d*_S_ ratios for the six pairs of duplicated genes (Table S4) and determined they were all less than one. This suggested the duplicated gene pairs experienced a purifying selection process (Goldman and Yang 1994).

### Expression analysis of 18 selected *Gossypium arboreum TIFY* genes

**Fig. 4:**
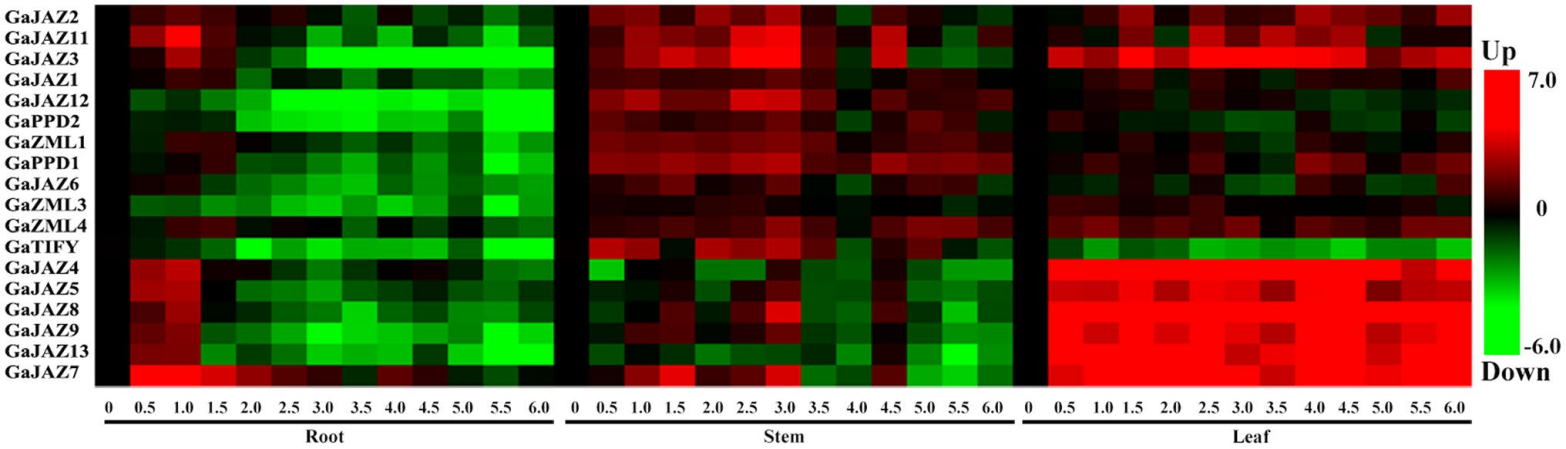
Expression levels of the 18 selected *TIFY* genes according to quantitative reverse transcription polymerase chain reaction

Because *TIFY* genes have a pivotal role in the JA signaling pathway, we analyzed the expression of 21 *G. arboretum TIFY* genes in roots, stems, and leaves under drought stress conditions (i.e., 17 % PEG 6000) to determine their functions. Among the 21 genes, 18 (86 %) were expressed in at least one of the three tissues (Fig. 4). Expression of the other three genes was not expressed according to the qRT-PCR analyses.

The expression levels of seven *GaJAZ* genes (i.e., *GaJAZ*3, *GaJAZ4*, *GaJAZ5*, *GaJAZ7*, *GaJAZ8*, *GaJAZ9*, and *GaJAZ13*) were highest in leaves at all time points. Treatment with 17 % PEG 6000 resulted in high transcript levels that peaked 1 h after the initiation of drought stress. However, expression levels tended to gradually decrease in the roots. In stems, transcripts of four *GaJAZ* genes (i.e., *GaJAZ2*, *GaJAZ3*, *GaJAZ11*, and *GaJAZ12*), *GaPPD1*, and *GaTIFY* peaked at 4 h after PEG treatment. Additionally, *GaJAZ7* was the most highly expressed gene in all tissues. Overall, the JAZ subfamily members exhibited the highest expression levels in the three analyzed tissues, suggesting *GaJAZ* genes are important for responses to drought stress.

### *TIFY* gene responses to drought conditions

**Fig. 5:**
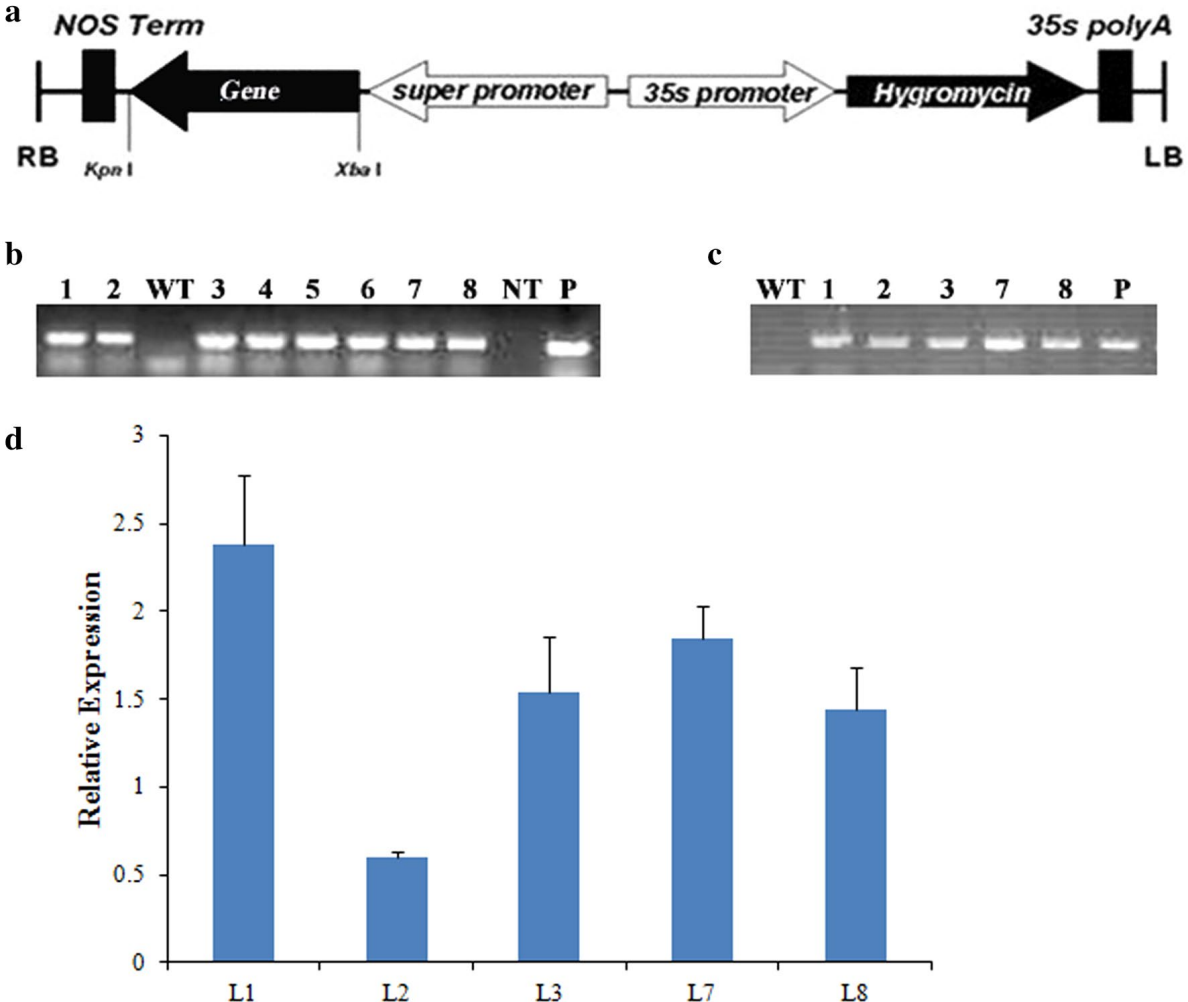
Plant transformation vector and the expression patterns of the *TIFY* genes in the transgenic lines. **a** Schematic representation of the T-DNA region of the pSuper1300-*GaJAZ5* binary vector. **b** PCR analysis of the selected marker gene. *WT* wild-type, *NT* non-template, *P* plasmid, *L1, L2, L3, L7, L8* transgenic lines expressing *GaJAZ5.* **c** Genomic PCR analysis of the *GaJAZ5* gene. **d** *GaJAZ5* expression patterns in transgenic plants. Values are presented as the means of three replicates. *Error bars* indicate the standard deviations. The histone gene was used as an internal control to normalize gene expression levels

A schematic representation of a 35S-*TIFY* gene construct is presented in Fig. 5a. The successful transformation of *A. thaliana* plants with the *TIFY* constructs was verified by PCR analyses of marker and target genes. Five independent *GaJAZ5* single-copy transgenic lines were generated, and analyzed by genomic PCR to confirm transformation and over-expression (Fig. 5b, c). The *GaJAZ5* expression levels varied among the transgenic lines, indicating the gene was successfully introduced into the transformed plants (Fig. 5d).

**Fig. 6:**
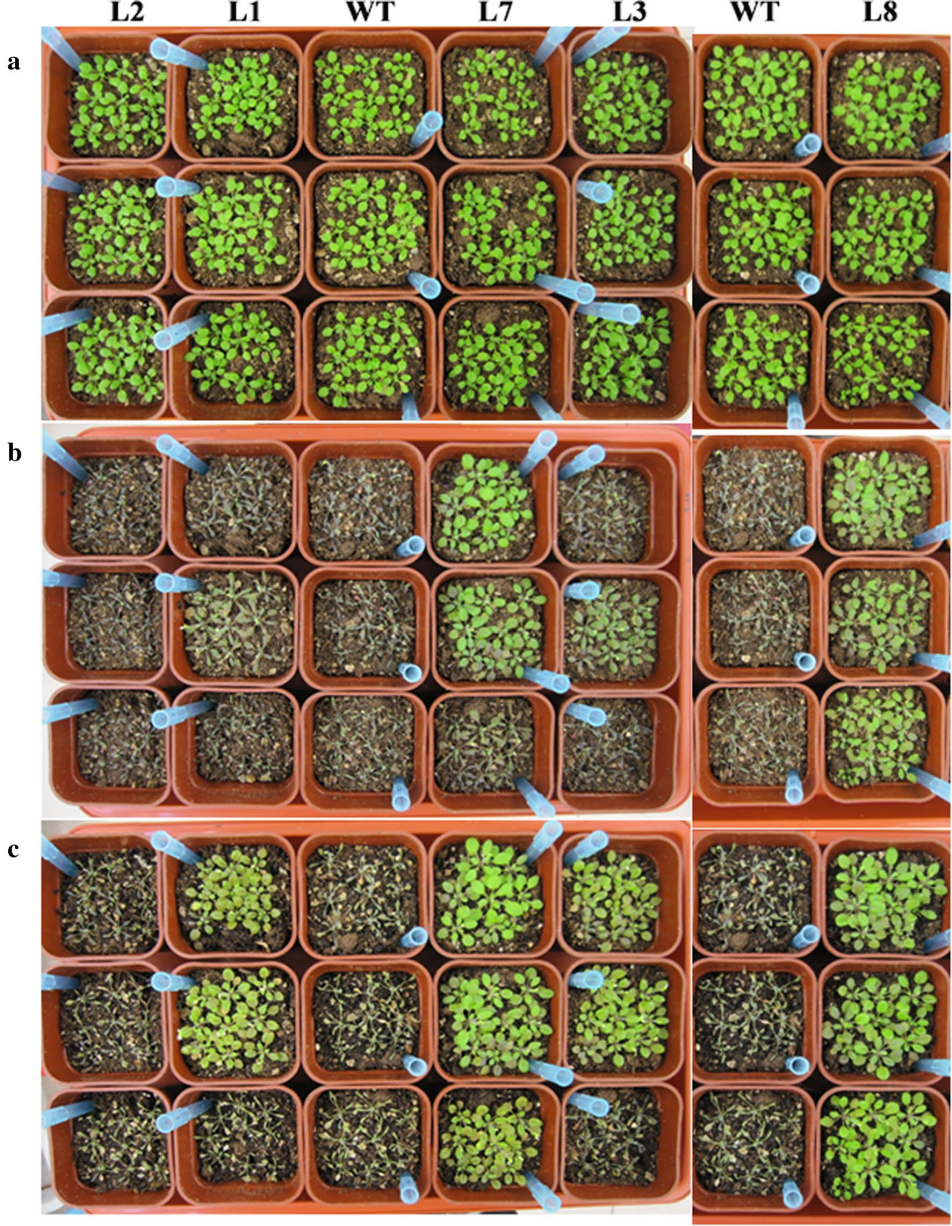
Drought tolerance assay. **a** Transgenic and wild-type (WT) plants cultured in sufficiently watered soil. **b** Transgenic and WT plants cultured in soil without water for 17 days. **c** Transgenic and WT plants cultured in soil after rewatering

To test whether *GaJAZ5* influenced drought resistance, a water loss assay was completed. After withholding water for 17 days, some of the WT plants wilted and died, while only a few *GaJAZ5* transgenic plants wilted. After plants were rewatered, the transgenic plants recovered from the drought conditions much better than the WT plants (Fig. 6). These results suggested that *GaJAZ5* affects drought tolerance.

**Fig. 7:**
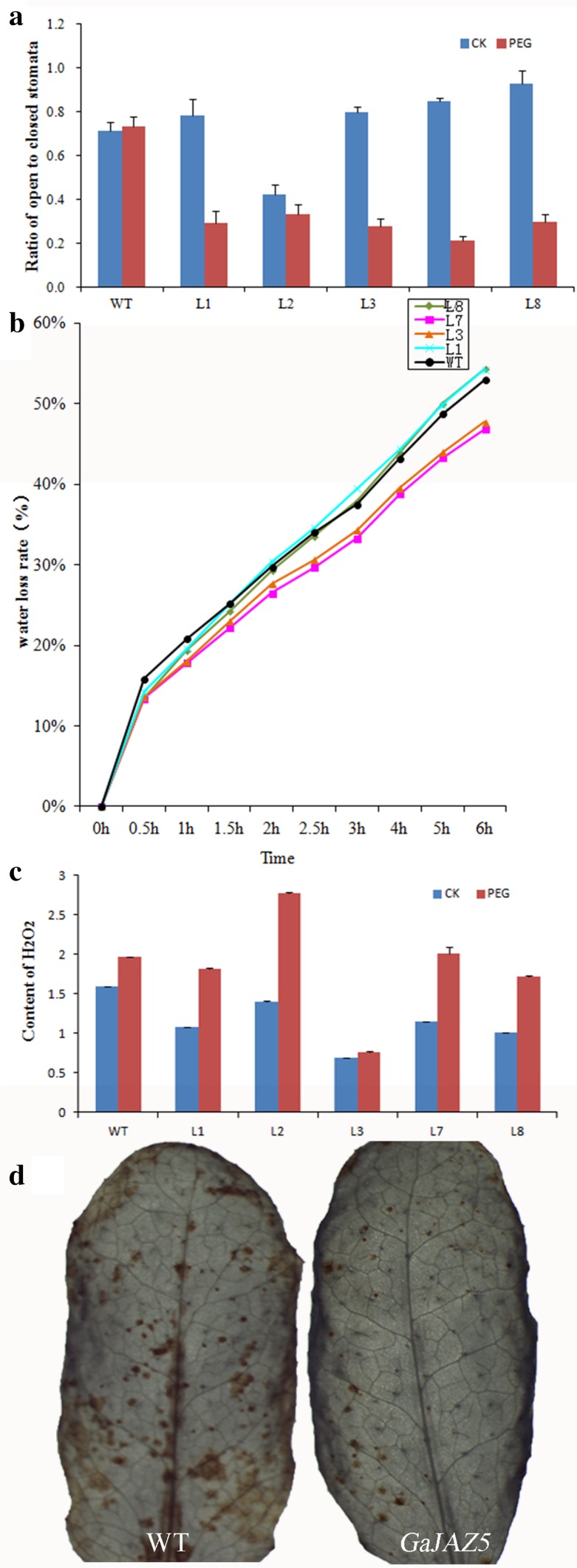
Biochemical and physiological assays involving *GaJAZ5*-expressing transgenic plants. **a** Ratio of open to closed stomata. **b** Rates of water loss in transgenic and wild-type plants. **c** H_2_O_2_ concentrations in transgenic lines. **d** Histochemical staining to detect H_2_O_2_ production. *OX* over-expression

The rate of water loss was investigated to further characterize how *GaJAZ5* influences water stress tolerance through the maintenance of high relative water content and a reduced rate of water loss. Because of their smaller stomatal apertures (Fig. 7a), the transgenic *A. thaliana* plants over-expressing *GaJAZ5* retained more water than the WT plants following treatment with 10 % PEG 6000. Additionally, the in vitro leaf water loss rate in transgenic lines 3 and 7 was lower than that of WT plants (Fig. 7b).

Under normal conditions, the H_2_O_2_ levels in all *GaJAZ5*-expressing lines were lower than those of the WT plants. Following treatment with 10 % PEG 6000, the H_2_O_2_ levels of three lines (especially transgenic line 3) were lower than those of the WT plants (Fig. 7c). This indicated that WT plants accumulated more H_2_O_2_ than the *GaJAZ5*-expressing transgenic plants after treatment with 10 % PEG 6000 (Fig. 7d).

## Discussion

### Importance of *TIFY* genes

Survey data indicated drought-affected environments will seriously threaten sustainable agricultural production of crops (http://www.isaaa.org/). Therefore, it is important to identify genes that promote high water use efficiency and that are suitable for plants in desert areas. Cotton is one of the most economically important crops worldwide (Kalivas et al. 2011). *Gossypium arboreum* (diploid) is drought tolerant, and a better candidate for studying drought tolerance-related genes than *G. hirsutum* (tetraploid) (Chen et al. 2015). Jasmonic acid regulates plant defense activities by inducing the expression of *TIFY* genes, which forms part of a negative feedback loop biosynthesis pathway and a signal transduction pathway that controls JAZ protein levels in response to JA and environmental stress (Song et al. 2014). *JAZ* genes are well-characterized members of the *TIFY* family with roles in JA-mediated processes, and thus are valuable for stress tolerance research.

### Phylogenetic analysis and classification of the *TIFY* gene family

TIFY homologs are only present in terrestrial plants, and not in green algae or other non-photosynthetic eukaryotes (Vanholme et al. 2007). This suggests that the *TIFY* family may have originated after aquatic plants evolved to survive on land. Comparative genomic analyses involving different taxa are useful for studying the structure, function, and/or evolution of genomes from uncharacterized taxa (d’Alencon et al. 2010). The *TIFY* family genes in *G. raimondii* and *G. arboreum* are highly conserved, but some *G. arboreum* genes were lost as the species evolved (Fig. 2). Both *G. raimondii* and *G. arboreum* underwent cotton-specific whole-genome duplication events approximately 16.6 million years ago. The modern allotetraploid *G. hirsutum* species resulted from hybridizations of two (A or D diploid) ancestral species approximately 1.5 million years ago (Li et al. 2015). All *TIFY* family members in the analyzed species contained four subfamilies, indicating they may have evolved similarly. Thus, we predicted target gene functions and/or evolutions using model plants such as *A. thaliana*. Previous studies demonstrated that eight *AtJAZ* genes (i.e., *AtJAZ1*, *AtJAZ2*, *AtJAZ5*, *AtJAZ6*, *AtJAZ7*, *AtJAZ8*, *AtJAZ9*, and *AtJAZ10*) were responsive to JA (Thines et al. 2007; Chung et al. 2009). Additionally, other aspects of *A. thaliana TIFY* gene activities, including alternative splicing (Chung et al. 2009) and interactions with MYC2 (Chini et al. 2007), were systematically analyzed to predict *TIFY* genes based on *A. thaliana* syntenic orthologs.

### Conservation and divergence: tandem and segmental duplications contributed to the expansion of the *TIFY* gene family

We detected two sequences with the same Garb number, and another sequence that had two Garb numbers (Zhang et al. 2013). Nevertheless, we identified 21 *TIFY* genes in the *G. arboreum* by motif analysis and subsequent molecular cloning, and determined that the cotton *TIFY* gene family is one of the largest *TIFY* families reported to date. Preliminary analyses revealed that gene duplication and subsequent divergence events were the main contributors to evolutionary momentum (Ohno et al. 1968; Chothia et al. 2003). Usually, the criteria for inferring a gene duplication event are as follows: the length of the alignment sequence covers ≥80 % of the longest gene, and the similarity of the aligned regions is ≥70 % (Gu et al. 2002; Yang et al. 2008). Two or more identical genes located on the same chromosome result from tandem duplications, whereas gene duplications between different chromosomes are designated as segmental duplications (He et al. 2012). The *TIFY* genes of *A. thaliana* and maize have undergone both segmental and tandem duplications, contributing to the expansion of the *TIFY* gene family. Tandem and segmental duplications of *TIFY* genes have also occurred in rice (Melotto et al. 2008) and grapevines (Zhang et al. 2012). Our study indicates that the *G. arboreum TIFY* genes are similar to those of *A. thaliana*, with tandem and segmental duplication events occurring in both species. Gene duplication followed by functional diversification (i.e., evolutionary changes in expression patterns) has played a vital role in driving the evolutionary processes that increase fitness to new environments (Conrad and Antonarakis 2007; Conant and Wolfe 2008). Future research may affect the topology of the phylogenetic tree in terms of the order of evolutionary distances for different members of the cotton *TIFY* family. Therefore, more information, especially regarding the chromosomal distribution of *TIFY* genes, is needed to accurately determine the evolutionary relatedness among these genes.

### *TIFY* gene responses to drought conditions

Transcriptome data suggested that *TIFY* gene family members affect drought tolerance (Zhang et al. 2012). As determined by qRT-PCR analysis, the *GaJAZ* genes were more highly expressed in the three analyzed tissues than the other groups of *TIFY* genes. This indicates the JAZ subfamily might have a vital role in regulating drought tolerance in cotton. Therefore, we used *GaJAZ5* in the drought assay.

When exposed to adverse environmental conditions, the stomata must close for plants to survive (Nauš et al. 2010). In this study, the stomatal apertures were smaller in the transgenic plants than in the WT controls. Stomatal closure reduces water loss, particularly in plants that have been exposed to water stress conditions caused by high solute concentrations (Wu et al. 2015) The observed reduced rate of water loss in transgenic plants might be caused by decreases in the size of the stomatal aperture. Additionally, compared with the WT plants, the *GaJAZ5*-expressing lines accumulated less H_2_O_2_, which may have protected cells from being damaged by drought or other stresses. During exposure to stress, increases in the metabolism of active oxygen species lead to the accumulation of H_2_O_2_ in plants. The accumulated H_2_O_2_ can directly or indirectly oxidize intracellular proteins, nucleic acids, and other biological macromolecules, as well as damage the cell membrane and accelerate cell collapse. Therefore, there is a close relationship between H_2_O_2_ content in plant tissues and plant responses to stress. The *GaJAZ5*-expressing lines responded better to drought conditions than the WT plants. The next step is to mimic the effects of drought through gene silencing or knockout.

Compared with the WT plants, the *GaJAZ5*-expressing lines were more drought resistant. Additionally, increased lateral root abundance and root hair density in *GaJAZ5*-expressing lines treated with methyl jasmonate can increase the total root surface area, which has implications for absorption of water and/or nutrients (Fig. S3). Following exposure to drought conditions or methyl jasmonate, JAZ proteins degrade and release transcription factors that can then increase the expression of downstream genes affecting drought resistance. The identities of the relevant *G. arboreum* transcription factors and target genes are currently unknown. Our future studies will focus on characterizing the mechanism of drought resistance and determining the relationship between drought stress and JA.

## Conclusions

The *TIFY* gene family has been extensively studied in model plant species such as Arabidopsis, but there has been a lack of systematic analysis of *TIFY* family genes in cotton, especially in the A genome species *G. arboreum*. Here, we identified and compared the *TIFY* gene family members of the cotton species, *G. raimondii* and *G. arboreum* and *G. hirsutum*. Through evolutionary analysis, we explored the expansion and functional divergence of these *TIFY* genes. The *TIFY* genes likely experienced tandem and segmental duplication events, similar to the corresponding *A. thaliana* genes. Gene expression profiles revealed the *GaJAZ* genes have important functions during drought responses. Additionally, the results of the drought tolerance assays and qRT-PCR analyses of *GaJAZ5* expression suggest that the JAZ subfamily may be involved in conferring drought tolerance to plants. Although, this study focused mainly on cotton *TIFY* genes, the relatively high conservation of orthologous *TIFY* family genes in various cotton interspecies indicates further comparative genomic analyses of other members of the family *Malvaceae* are warranted. These additional studies will help generate useful germplasm resources, which will be relevant for the breeding of drought-tolerant cotton species.

## **Electronic supplementary material**

Below is the link to the electronic supplementary material.

Supplementary material 1 (XLS 480 kb)

Supplementary material 2 (TIFF 8580 kb)

Supplementary material 3 (TIFF 5358 kb)

Supplementary material 4 (TIFF 321 kb)

Supplementary material 5 (XLS 23 kb)

Supplementary material 6 (XLS 21 kb)

Supplementary material 7 (XLS 26 kb)

Supplementary material 8 (XLS 20 kb)

## Abbreviations

bHLH: Basic helix-loop-helix
PEG: Polyethylene glycol
JA: Jasmonic acid
SCF^COI1^: Ubiquitin ligase complex
ZIM: Zinc finger protein expressed in inflorescence meristem
d_S_: Synonymous
d_N_: Non-synonymous
JAZ: Jasmonate ZIM domain
Bp: Base pair
BLAST: Basic local alignment search tool
PCR: Polymerase chain reaction
qRT-PCR: Quantitative reverse transcription PCR
WT: Wild-type
