# Supplementary figures and images for: Genome-wide identification and functional analysis of the TIFY gene family in response to drought in cotton
Source: Mol Genet Genomics. 2016 Sep 17;291(6):2173–87. doi: 10.1007/s00438-016-1248-2 (PMC5080297; doi:10.1007/s00438-016-1248-2)

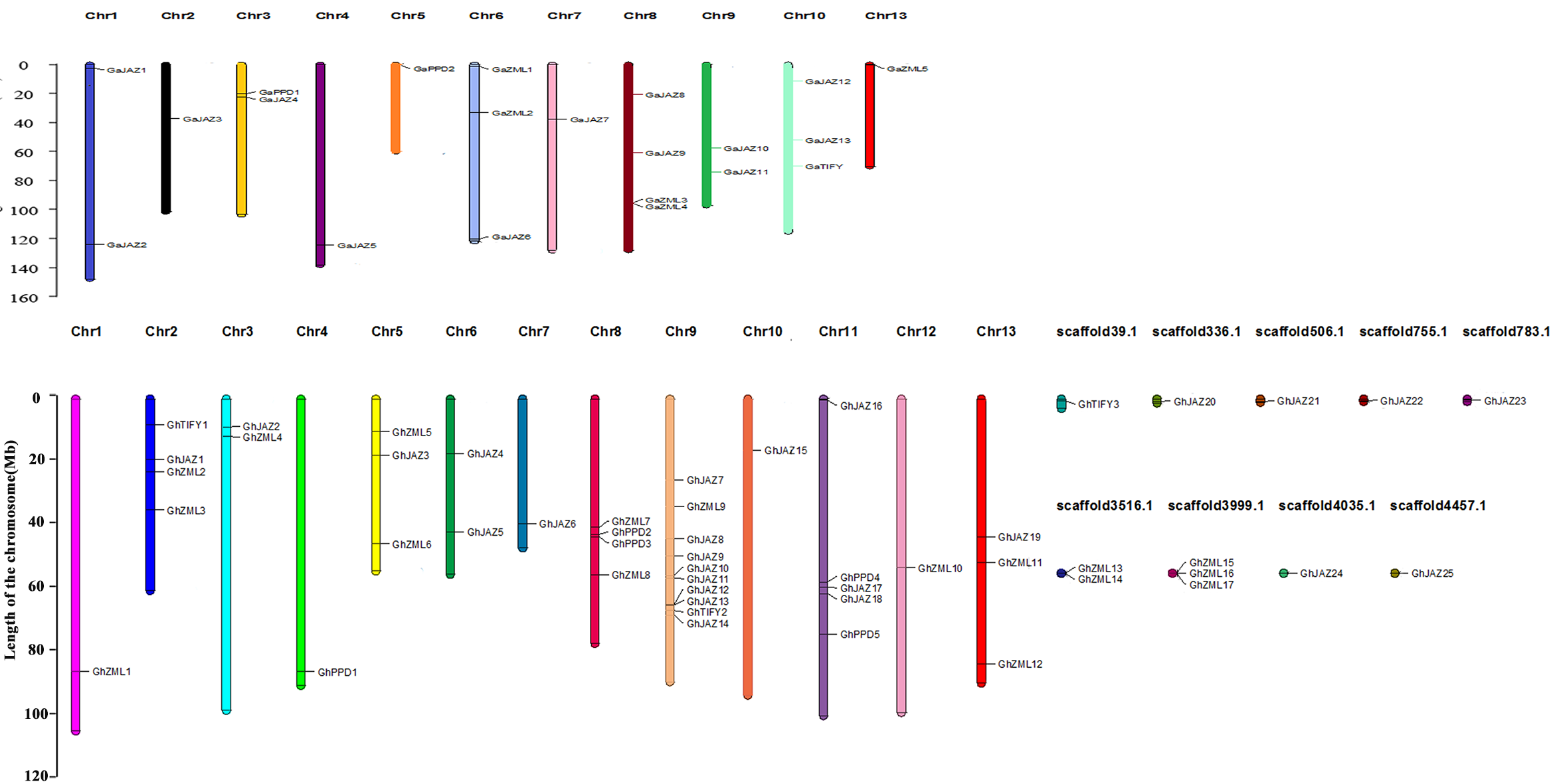

Supplement: Supplementary file 2 — Supplementary material 2 (TIFF 8580 kb) [file 438_2016_1248_MOESM2_ESM.tif]

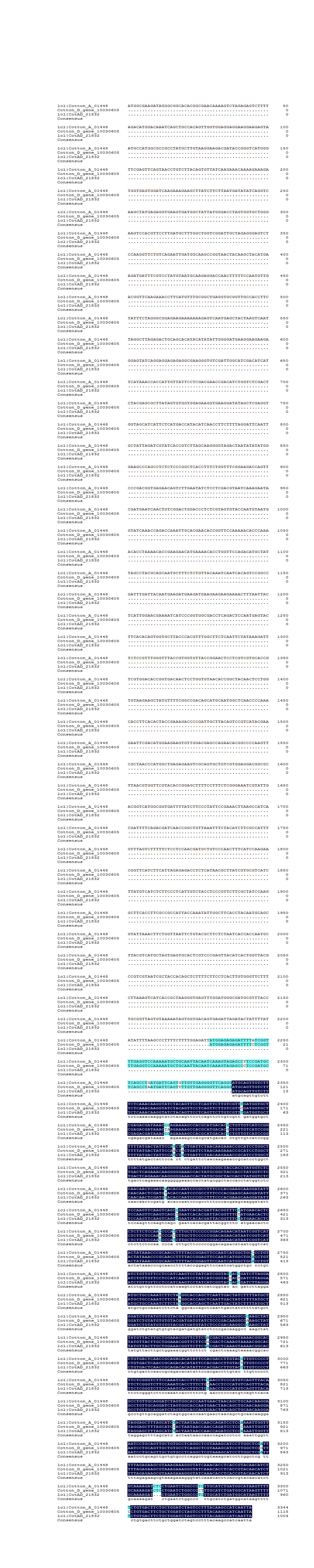

Supplement: Supplementary file 3 — Supplementary material 3 (TIFF 5358 kb) [file 438_2016_1248_MOESM3_ESM.tif]
